# Point-of-care testing in private pharmacy and drug retail settings: a narrative review

**DOI:** 10.1186/s12879-023-08480-w

**Published:** 2023-08-23

**Authors:** Justine Tin Nok Chan, Van Nguyen, Thuy Ngan Tran, Nam Vinh Nguyen, Nga Thi Thuy Do, H. Rogier van Doorn, Sonia Lewycka

**Affiliations:** 1 Fitzwilliam College, University of Cambridge, Cambridge, UK; 2 Doctor of Medicine Programme, Duke National University of Singapore (NUS) Medical School, Singapore, Singapore; 3 Oxford University Clinical Research Unit, Hanoi, Vietnam; 4 Family Medicine and Population Health (FAMPOP), Faculty of Medicine and Health Sciences, University of Antwerp, Antwerp, Belgium; 5 Centre for Tropical Medicine and Global Health, Nuffield Department of Medicine, University of Oxford, Oxford, UK

**Keywords:** Rapid diagnostic tests, Drug resistance, Communicable diseases, Pharmacies, Feasibility studies

## Abstract

**Background:**

Point-of-care testing (POCT) using rapid diagnostic tests for infectious disease can potentially guide appropriate use of antimicrobials, reduce antimicrobial resistance, and economise use of healthcare resources. POCT implementation in private retail settings such as pharmacies and drug shops could lessen the burden on public healthcare. We performed a narrative review on studies of POCTs in low- and middle-income countries (LMICs), and explored uptake, impact on treatment, and feasibility of implementation.

**Methods:**

We searched MEDLINE/PubMed for interventional studies on the implementation of POCT for infectious diseases performed by personnel in private retail settings. Data were extracted and analysed by two independent reviewers.

**Results:**

Of the 848 studies retrieved, 23 were included in the review. Studies were on malaria (19/23), malaria and pneumonia (3/23) or respiratory tract infection (1/23). Nine randomised controlled studies, four controlled, non-randomised studies, five uncontrolled interventions, one interventional pre-post study, one cross-over interventional study and three retrospective analyses of RCTs were included. Study quality was poor. Overall, studies showed that POCT can be implemented successfully, leading to improvements in appropriate treatment as measured by outcomes like adherence to treatment guidelines. Despite some concerns by health workers, customers and shop providers were welcoming of POCT implementation in private retail settings. Main themes that arose from the review included the need for well-structured training with post-training certification covering guidelines for test-negative patients, integrated waste management, community sensitization and demand generation activities, financial remuneration and pricing schemes for providers, and formal linkage to healthcare and support.

**Conclusion:**

Our review found evidence that POCT can be implemented successfully in private retail settings in LMICs, but comprehensive protocols are needed. High-quality randomised studies are needed to understand POCTs for infectious diseases other than malaria.

**Supplementary Information:**

The online version contains supplementary material available at 10.1186/s12879-023-08480-w.

## Background

### Antimicrobial stewardship

Antimicrobial resistance (AMR) is a critical issue requiring effective antimicrobial stewardship (AMS) [1]. AMS can prevent antibiotic overuse, misuse, and abuse [1] and reduce drug resistance [2], costs, and hospital stays [3, 4]. This is particularly important for low- and middle-income countries (LMICs), who suffer a greater AMR [4] and infectious disease burden [5]. Well-developed stewardship measures applicable to LMICs are thus a research priority.

### Point-of-care testing

Diagnostic tests to support appropriate treatment and prescribing are a key component of AMS programmes. However, lower-level healthcare settings in LMICs have limited laboratory and diagnostic capacity. Point-of-care testing (POCT), in which patient specimens are analysed outside of a clinical laboratory, at the site of patient care, by staff who have not been formally trained in laboratories, offers a means of reaching more patients with diagnostic services [6]. POCT represents a promising avenue for enhancing antimicrobial stewardship. For example, C-reactive protein (CRP) testing provides real-time assessment of likelihood of bacterial infection, reducing antibiotic prescribing in primary care [7–10]. In a review, 44% of patients who received CRP tests were prescribed antibiotics at initial consultations for upper respiratory tract infections (RTIs), compared to 63% of those untested [11]. More specific POCTs for particular infectious diseases like malaria can also guide appropriate antimicrobial therapy, thereby contributing to reduced disease burden and resistance, and enabling rapid diagnosis for a disease that previously only relied on clinical diagnosis. A malaria POCT test-and-treat strategy in Zambia reduced paediatric prevalence of malaria by 17% [12]. Moreover, POCTs can help reduce unnecessary use of drugs, staff, and equipment [13], lowering costs. For example, *Helicobacter pylori* screening reduces the number of patients referred for endoscopy [14].

### Implementation in private settings

POCT in private retail settings such as pharmacies and drug shops is particularly promising for LMICs [15], where trained workforce and infrastructure are lacking [16], as they are independent of expensive, centralised laboratories [17]. POCTs are easy to perform, interpret, and transport [18]. Although rapid diagnostics are available in large public hospitals, with median availability of malarial diagnostics reaching 91.6% in 10 LMICs [19], these services are often overloaded [20]. In contrast, primary care tends to lack diagnostics, with only 19.1% median availability [19]. Adding additional diagnostic services in hospitals and primary care would burden the national budget. Hence, POCTs in private settings could make diagnostic services more accessible.

Moreover, pharmacies are an ideal checkpoint for antimicrobial use, as regulations around dispensing antimicrobials are poorly enforced in LMICs, with frequent over-the-counter non-prescribed antimicrobial sales [21]. By detecting or ruling out infection, POCTs can help providers recommend appropriate treatment [22–27]. Their use in pharmacies and drug shops can reduce unnecessary treatment and improve care-seeking behaviours [25–28], while still providing revenue from test sales.

### Aim

In this study, we reviewed evidence for implementation of POCTs for infectious diseases in private retail settings in LMICs, to inform future studies and policy design.

## Methods

### Search strategy

This review was structured with reference to the Scale for the Assessment of Narrative Review Articles (SANRA) [29] and Preferred Reporting Items for Systematic Reviews and Meta-Analyses (PRISMA) [30]. We searched PubMed/Medline on 26/06/2023, using MeSH headings and synonyms for ‘infectious disease’, ‘rapid diagnostic testing’, and ‘pharmacy’ (full search terms Additional file 1). A manual search of references from other studies was also conducted to include relevant studies. No limit on date of publication was imposed on studies for inclusion.

Inclusion criteria were developed referencing population-intervention-comparison-outcomes (PICO):

- Participants: pharmacies and private retailers in LMICs
- Interventions: implementation of staff-performed POCT
- Comparisons: N/A
- Outcomes: feasibility and impact of implementation

Non-private or non-interventional studies (reporting only test accuracy, hypothetical or modelling studies, lacking actual implementation); or not on infectious diseases, were excluded.

Two independent investigators selected papers for full-text screening using Covidence, resolving conflicts by discussion. Investigators designed an abstract screening tool [31] (Additional file 2) and randomly selected 20 papers for standardisation of screening.

### Outcomes

A data extraction table was created using Google Sheets 2023. Two authors independently performed extraction of study characteristics, methods, and outcomes:

- Uptake: proportion of treatment-seeking patients receiving POCT out of total population studied
- Positivity: proportion of patients receiving a POCT who tested positive
- Treatment provision: proportions of treatment-seeking patients and of patients not tested receiving intended treatment(s)
- Adherence to POCT results: proportions of POCT-negative patients not receiving intended treatment(s) and of POCT-positive patients receiving intended treatment(s)
- Referrals: proportion of patients referred elsewhere
- Test accuracy: sensitivity, specificity, or positive predictive value of test (if available)
- Safety and accuracy of performance of test: proportion of providers safely and correctly performing, interpreting and disposing of POCT (if available)
- Recommended/median POCT retail price (if available)
- Opinions of providers/customers on POCT

Proportions were expressed as percentages. Individual outcomes were chosen or calculated from cluster data. Additional characteristics extracted were training length/content, supervision, demand-generation activities, referral, and guidelines for those who tested positive or negative on the POCT.

### Quality assessment

Article quality was checked against seven of ten relevant features published by the Consensus Working Group of the Joint Programming Initiative on Antimicrobial Resistance: randomized design; use of controls; multi-centre study design; sustainability of intervention (> 12 months); sample size calculation (where relevant); prospective design; and correction for confounding variables [32]. Funding was evaluated for conflicts of interest.

## Results

### Study selection

**Fig. 1:**
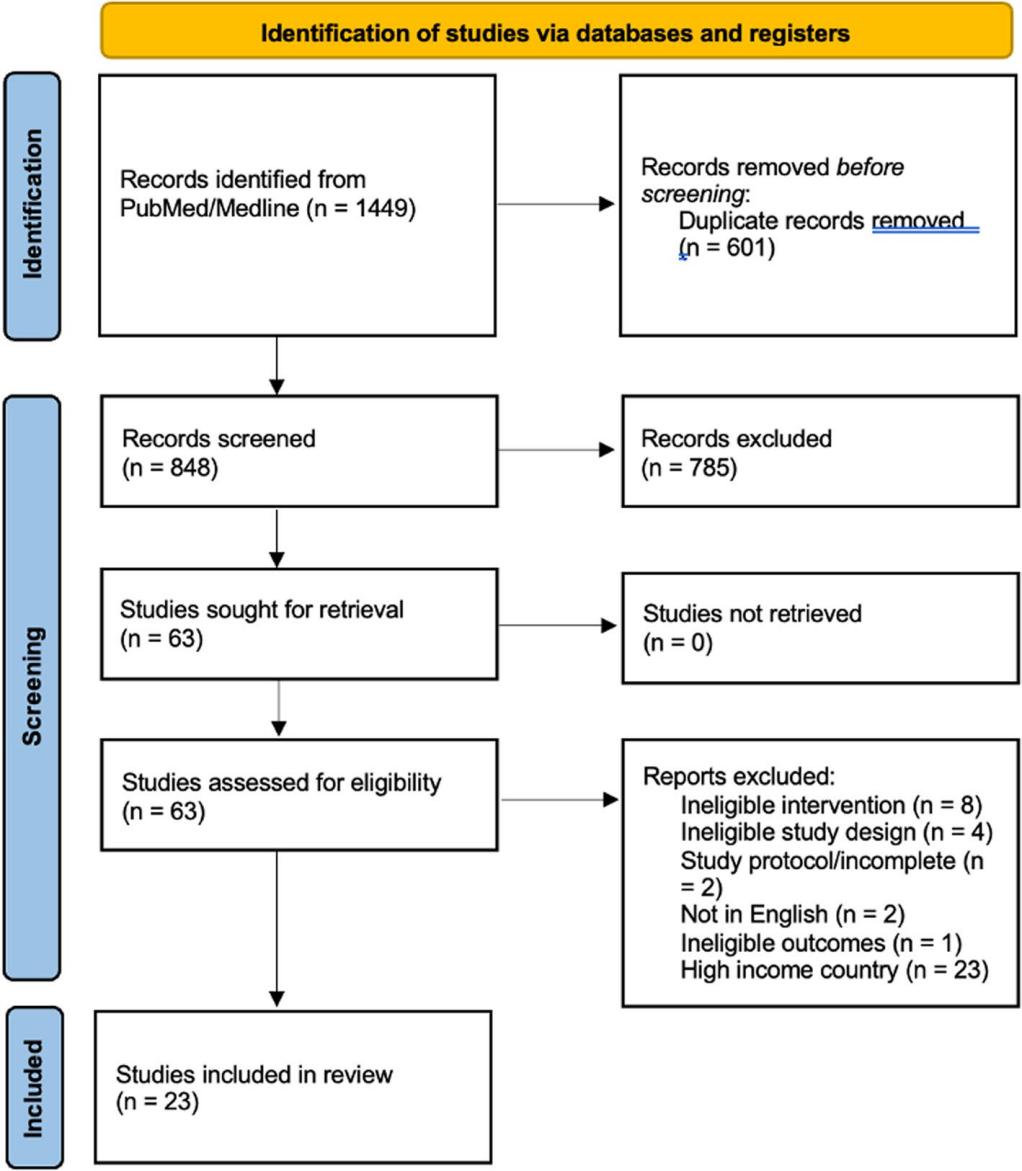
PRISMA flow diagram for studies analysed in this narrative review

From our search strategy, 848 titles were identified, of which 63 studies were screened in full (Fig. 1). 21 studies were excluded based on pre-defined inclusion/exclusion criteria. 19 studies in high-income countries were excluded after review to focus on LMICs. 23 studies were included, including one study detailing the policy implications from another study included in this review [33, 34].

### Study design and characteristics

Table 1 describes characteristics of the included studies. 19 studies were on malaria, two were on a mix of malaria and pneumonia, one was on paediatric fever management, and one was on respiratory tract infections of viral or bacterial aetiology. Study designs included nine randomised controlled studies, four controlled, non-randomised studies, five uncontrolled interventions, one interventional pre-post study, one cross-over interventional study and three retrospective analyses of RCTs. Hansen et al. (2017) was a cost-effectiveness analysis of Mboyne et al. (2015)’s malaria study [26, 34].

**Table 1:** Study information and characteristics

Ref no	First author	Country	Year performed	Year published	Length of study (months)	Type of study	Name of POCT	Targeted disease	Targeted pathogen or antibody	Patient sample used for test
[23]	Ansah	Ghana	2011 to 2013	2015	18	Clustered randomized (RCT)	CareStart Malaria HRP2 Pf	Malaria	Plasmodium falciparum	Blood
[35]	Audu	Ghana	2014	2016	6	Prospective cross-over study	Blue Aid Malaria Test Kit	Malaria	P. falciparum, Plasmodium vivax	Blood
[36]	Aung	Myanmar	2013	2015	6	Clustered randomized (RCT)	Malaria POCT (unnamed)	Malaria	P. falciparum	Blood
[24]	Awor	Uganda	2011 to 2012	2014	13	Controlled but non-randomised study	Malaria POCT (unnamed) and respiratory timers	Malaria and pneumonia	NA	Malaria: blood Pneumonia: breathing rate
[37]	Awor	Uganda	2011 to 2012	2015	13	Controlled but non-randomised study	Malaria POCT (unnamed) and respiratory timers	Malaria and pneumonia	NA	Malaria: blood Pneumonia: breathing rate
[38]	Cohen	Uganda	2011–2012	2012	13	Interventional study without controls	Care Start Malaria HRP2 Pf	Malaria	P. falciparum	Blood
[26]	Hansen	Uganda	2011	2017	12	Cost effectiveness analysis of an RCT (see Mboyne et al.)	Care Start Malaria HRP2 Pf	Malaria	P. falciparum	Blood
[39]	Hutchinson	Uganda	2010 to 2012	2015	22	Clustered randomized (RCT)	Malaria POCT (unnamed)	Malaria	NA	Blood
[40]	Hutchinson	Uganda	2010 to 2012	2017	22	Clustered randomized (RCT)	Malaria POCT (unnamed)	Malaria	NA	Blood
[41]	Ikwuobe	Nigeria	2012	2013	3	Controlled but non-randomised study	SD BIOLINE Malaria Antigen Pf	Malaria	P. falciparum	Blood
[42]	Kitutu	Uganda	2013 to 2015	2017	16	Controlled but non-randomised study	Care Start Malaria HRP2 Pf and respiratory timers	Malaria, pneumonia and bloody diarrhoea	P. falciparum	Malaria: blood Pneumonia: breathing rate
[43]	Kwarteng	Ghana	2013	2019	8	Interventional study without controls	Care Start Malraria HRP2 Pf	Malaria	P. falciparum	Blood
[44]	Maloney	Tanzania	2013 to 2014	2017	15	Clustered randomized (RCT)	ParaHIT Ag Pf POCTs	Malaria	P. falciparum	Blood
[34]	Mbonye	Uganda	2011	2015	12	Clustered randomized (RCT)	Malaria POCT (unnamed)	Malaria	NA	Blood
[33]	Mbonye	Uganda	2011	2015	See Mboyne 2015	Policy analysis of Mboyne (2015)				
[45]	Onwunduba	Nigeria	2022	2023	6	Cluster randomized trial (RCT)	CRP test kit from Zhuhai Encode Medical Engineering Co	Respiratory tract infections (RTI)	Viruses or bacteria causing RTIs	Blood
[46]	O' Meara	Kenya	2014–2015	2016	11	Factorial randomized (RCT)	Malaria POCT (unnamed)	Malaria	NA	Blood
[47]	Poyer	Kenya	2013–2016	2018	18	Interventional pre-post study	CareStart Malaria HRP2 (Pf)	Malaria	P. falciparum	Blood
[48]	Shelus	Uganda	2021	2023	3 months	Uncontrolled interventional trial	8 types of RDTs, most frequently: SD Bioline, SD Biosensor, Carestart, and First Response	Malaria	P. falciparum	Blood
[49]	Simmalavong	Laos	2008–2016	2017	108	Interventional study without controls	Malaria POCT (unnamed)	Malaria	NA	Blood
[50]	Soniran	Ghana	2019–2020	2022	14	Cluster randomized trial (RCT)	Malaria POCT (unnamed)	Malaria	NA	Blood
[51]	Sudhinaraset	Myanmar	2013	2015	6	Qualitative study of RCT	FIRST RESPONSER Malaria antigen pLDH/HRP2 combo card test	Malaria	NA	Blood
[52]	Thet	Myanmar	2019–2020	2021	3	Interventional study without controls	Malaria POCT (unnamed)	Malaria	P. falciparum	Blood

Ref no	Urban/Rural	Type and number of outlets included in study groups	Description of clientele served by private stores selling POCT included in study	Sharp box and/or gloves provided?	Length and content of provider training	Guidelines for patients that test positive	Guidelines for patients that test negative	Supervision frequency and method of private providers of POCT	Demand generation activities	Recommended retail price of POCT
[23]	Rural	24 communities with 1 to 5 chemical shops per community	Clients with fever or who requested antimalarials, who were not pregnant, > 6 months old, no severe disease, no prescription from health facility, in district for > = 28 days	Bins for disposal of sharps, reference charts for doses of artemisinin therapy	3 days on Ghana’s antimalarials policy, symptoms, indications for referral, blood sampling, blood safety, sharps usage, infection prevention, study protocol Intervention arm: extra 1 day, how to perform, interpret and manage negative POCTs, practise sessions	Encourage clients to purchase ACTs	Refer to nearby healthy facility or facility of choice	Recorded by seller on customised form which was subject to random checks by study authors; mystery clients	Community sensitization meetings and durbars (traditional community leaders)	NA
[35]	Mixed	6 private retail pharmacies in 3 different districts of the Ashanti region	1200 patients with fever or history of fever in past 48 h	No	Technique and usage of POCT	NA	NA	Recorded by pharmacy on reporting form, which was studied daily by principal researcher; Microscopy to confirm diagnoses	NA	NA
[36]	Rural	171 general retail stores, drug vendors, medical drug representatives	Households who had fever in last 3 weeks and taken antimalarials or had malaria symptoms, lived in an area where ACT was sold in private sectors	Antiseptic pad provided	Use, interpretation and safe disposal of POCT	Prescribe ACT	Refer to nearest health facilities	Arm 1: Monthly check-in visit, Arm 3: Bi-monthly intensive support visits with one-on-one discussions, information, education and communication	NA	Price subsidy for POCT resupply at $0.18/test
[24]	Rural	Intervention: 44 registered drug shops Control: 40 registered drug shops	Caretakers and children (< 5y/o and febrile) who sought care in drug shop or lived in participating districts	No	5 days of 2 drug shop attendants per drug shop on how to use POCT for fevers and respiratory timer for coughs, dispense pre-packaged drugs, via clinical sessions	Dispense recommended treatment of ACTs (malaria) and amoxicillin (pneumonia)	NA	Direct observation by field supervisor (nurse)	Branding of drug shops, communicating with caretakers of children, information on care-seeking provided at markets, public gatherings and radios	Free POCTs; Subsidised drugs: ACTs, amoxicillin, oral rehydration solution, zinc sulfate at 50–80% mark-up, selling at USD 0.38
[37]	Rural	Intervention: 44 registered drug shops Control: 40 registered drug shops	Caretakers and children (< 5y/o and febrile) who sought care in drug shop or lived in participating districts	No	5 days of 2 drug shop attendants per drug shop on how to use POCT for fevers and respiratory timer for coughs, dispense pre-packaged drugs, via clinical sessions	Dispense recommended treatment of ACTs (malaria) and amoxicillin (pneumonia)	NA	Direct observation by field supervisor (nurse)	Branding of drug shops, communicating with caretakers of children, information on care-seeking provided at markets, public gatherings and radios	Free POCTs; Subsidised drugs: ACTs, amoxicillin, oral rehydration solution, zinc sulfate at 50–80% mark-up, selling at USD 0.38
[38]	Mainly rural	92 drug shops in 58 villages that offered POCTs after completing training and households in the selected villages	Households in 67 villages with at least 1 pharmacy	Gloves and sharps disposal box provided	2 days (adapted from a WHO-based organization) on POCT, administration procedures, results interpretation	No specific instruction was provided other than proceed as usual	No specific instruction was provided other than proceed as usual	Monthly monitoring visit with administrative record checks from wholesale distributors	NA	Shops bought it from wholesalers at an agreed US 0.20 and sold at shop’s discretion
[26]	Mainly rural	20 randomized clusters, with10 for each intervention arm	Population with a majority living in rural areas/farmers seeking care for fever	No	3 days on malaria case management, 1 extra day for intervention arm on POCT	Recommend ACT purchase	No ACT or other anti-malarials would be sold	Close support visit for first 3 months, lessened after	Community sensitization programs	POCTs provided to drug shops for free. Recommended retail price was $0.20
[39]	NA	Registered drug shop vendors, residents in area around drug shop who were clients or cared for clients, health workers in area	Participants who had been to the drug shop or cared for someone who had been	Blood slides and slide box, gloves, lancets, swabs and cotton wool provided	Both arms: 3 days on malaria, taking blood samples Intervention arm: 1 more day on POCTs	NA	NA	2 months of supervision of at least 3 supervisory visits; scaled back later with periodic contact; one more visit at 12 months	Roadside sign advertising POCT availability	Free
[40]	NA	59 registered drug shops	Participants who had been to the drug shop or cared for someone who had been	Blood slides and slide box, gloves, lancets, swabs and cotton wool provided	Both arms: 3 days on malaria, taking blood samples Intervention arm: 1 more day on POCTs	NA	NA	2 months of supervision of at least 3 supervisory visits; scaled back later with periodic contact; one more visit at 12 months	Roadside sign advertising POCT availability; community sensitization through Village Health Teams	Given for free and asked to sell at 0.20 USD
[41]	Suburban	Intervention: 1 pharmacy with sufficient anti-malarial sales per day (> = 23 per day) Control: 1 pharmacy	Patients with symptoms of malaria seeking malaria treatment (with an anti-malarial prescription or wanting to self-medicate with them)	No	How to conduct POCTs	Permit purchase of antimalarial	Pharmacist and patient would discuss to suspend anti-malarial treatment	NA	NA	NA
[42]	Mixed—Rural (6), Suburban (12), Urban (14 stores)	Intervention: 61 drug shops Control: 23 drug shops	Care seekers for children (< 5 y/o) with symptoms	No	Provision of information on workflow, information/education, communication on malaria, pneumonia, non-bloody diarrhoea treatments	Provide ACT (malaria) or amoxicillin DT (pneumonia)	Further evaluation and referral	Monthly supervision by supervisor trained in medicine, may be accompanied by district drug inspector and educator	Marking of intervention drug shops with posters, community sensitization campaign via radio talks	Free
[43]	Rural	3 pharmacy shops, 68 licensed chemical shops	Clients with fever or malaria signs/symptoms without signs of severe malaria	Gloves, disposal bins provided	1 week workshop on malaria treatment, POCT administration and counselling of results	Dispense ACT	Symptomatic treatment, return advice, withhold ACT	Bimonthly supervisory visits	NA	Free to drug shops. No recommended retail price
[44]	Rural	Intervention: 1 subsidised districts with 147 accredited drug dispensing outlets (ADDOs), 1 unsubsidised district with 115 ADDOs Control: 1 district	18 y/o customers seeking treatment for fever, suspected malaria, or trying to purchase anti-malarial	Gloves, sharps box provided	Six two-day trainings on recognising malaria, use of POCTs, and treatment	Prescribe ACT based on Artemether and lumefantrine treatment based on provided dosing reference chart	Referral of severely ill patients to nearest public health facility	Quarterly monitoring visits, during which dispenser was observed directly by study staff and shop conditions were evaluated	Storefront sign advertising malaria testing	Non-subsidised: 0.67 USD, Subsidised: < = 0.32 USD
[34]	Urban and periurban	59 drug shops	Febrile patients seeking treatment at drug shops	No	3 days for general malaria-related training Intervention arm: 1 extra day for POCT usage, blood slices preparation with extra training on communication skills to explain POCT diagnostics	Prescribe ACT	POCT-negative cases with fever: consider referral, no ACT/antimalarials would be sold	2-month period of supervision with at least 3 supervisory visits, 12-month follow-up	Signs placed outside for advertisement, community sensitization programs prior to trial	POCTs provided for free. Priced at $0.20
[33]										
[45]	Urban	20 private community pharmacies (10 intervention, 10 control)	Simulated clients who were trained to visit the pharmacy and complain of particular RTI symptoms	NA	Length was not described Training on use of CRP test kits and distinguishing viral and bacterial etiologies based on test kits	Advised not to dispense antibiotics to those with CRP < 30 mg/l, to use clinial judgments for CRP levels 30 ≤ CRP < 100 mg/l, dispense antibiotics if CRP ≥ 100 mg/l, but not discouraged from using professional judgement regardless of CRP results	Mystery clients visiting the pharmacies and presenting with RTI-like symptoms	NA		RDTs were provided and pharmacies were asked to charge < 1 USD per test
[46]	Rural	11 medicine shops	Any individual < 1 years old with malaria-like illness or symptoms during past 24 h	No	3 days on how to perform POCT	NA	NA	Field visits 1 week after enrolment of patients	NA	Yes, either free or $0.50 depending on group
[47]	Mixed	317 outlets (142 private health facilities and 175 pharmacies)	Adults seeking treatment for fever for themselves or on behalf of someone else	Gloves and sharps box provided	Training on malaria epidemiology, POCT procedure, case management for positive and negative test results	Prescribe ACT	Private health facilities—further investigatino, pharmacies—referral to health facility	routine supportive supervision visits	Radio, printed materials, small group sessions to highlight that not all fevers are malaria	0.80 USD for POCT from hospital pack, 1 USD for POCT single pack
[48]	Rural	46 drug shops in 20 villages	Clients visiting participating drug shops reporting fever or purchasing antimalarials for themselves or other individuals	Not specified but given "materials to safely collect blood samples and dispose of waste"	90 min training on study and RDT procedures	NA	Na	NA	NA	NA
[49]	Urban	317 pharmacies, 30 clinics	NA	NA	NA	NA	NA	NA	NA	NA
[50]	Rural	12 over-the-counter medicine sellers (7 intervention, 5 control)	Children under 10 years old with fever or suspected malaria in nearby households	NA	2 day training on malaria management, treatment and follow-up	Provide ACT	NA	Quarterly supportive visitis during which skills from training were reinforced and technical guidance was provided	Community health workers and town criers engaged to carry out sensitization on malaria highlighting the imporance of malaria testing before treatment at religious venues and community durbars	0.44 USD
[51]	Rural	30 informal providers	NA	NA	NA	NA	NA	NA	NA	NA
[52]	Urban	80 malaria drug shops for quantitative, 65 of these for qualitative interviews	Patients who were febrile and seeking care and drug shops	No	1 day on use of POCT, national guidelines on treatment, stock and waste management, counselling, reporting to national centres	“Effective and quality” antimalarial drugs	Antipyretics, analgesics, medicines, but not antibiotics	NA	NA	NA

Studies lasted between three [41, 52, 53] and 108 months [49]. Of the studies where the setting of the study was described, nine were in rural areas [23, 24, 26, 36–38, 43, 44, 46, 50, 51, 53], five in urban/suburban regions [33, 34, 41, 45, 49, 52], and two were in a mix of both [42, 47]. The number of outlets investigated per study ranged from two [41] to 317 [49].

Only eight studies described the consumable equipment they provided to the private retailers, like antiseptic pads [36], free gloves and sharps disposal [23, 38, 44, 47], or bins [39, 40, 43]. 20 studies implemented training for providers, covering study protocol, signs/symptoms, evaluation, and diagnostic criteria. Intervention arms also received training on POCT administration, interpretation, and disposal. Training lasted between 90 minutes [53] and six 2-day sessions [44].

For malaria, guidance for patients testing positive were provision of the appropriate medication, such as ACTs for malaria [23, 24, 26, 34–37, 41–44, 47, 52]. For other POCTs, patients satisfying disease criteria based on the test results were supposed to be prescribed amoxicillin for pneumonia [24, 37, 42], antibiotics for CRP levels greater than or equal to 100 mg/l [45], or zinc sulfate tablets for non-bloody diarrhoea [42]. For negative test results, clients were often referred to formal care [23, 36, 42, 47], especially if there was fever [34]. Sellers were to recommend stopping antimalarials [41] or against their purchase [24, 26, 34, 37, 43], or in cases of CRP less than 30 mg/l, to not dispense antibiotics [45]. Kwarteng et al. (2019) provided symptomatic treatment [43]. In Onwunduba et al. (2022), cases with CRP of intermediate levels between 30 mg/l to 100 mg/l, sellers were asked to use their professional judgment to decide on prescription of antibiotics [45].

Demand-generation activities were paper-based like roadside posters, media-based like newspapers or radio, and/or verbal promotions during healthcare consultations or durbars held by traditional community leaders [23].

### Methods of assessing outcomes

Table 2 summarises study methodologies.

**Table 2:** Studies’ methods for assessing outcomes

				**STUDY METHODS**				
**Ref no**	**First author**	**Year published**	**Targeted disease**	**Safety and accuracy of POCT administration**	**Accuracy of POCT**	**POCT testing: uptake, positivity**	**Treatment decision**	**Retail price method**
[23]	Ansah	2015	Malaria	Mystery clients were directly observed weekly in 1st month and for 1 more week halfway through trial; regular quality control by sampling test kits using positive blood samples	NA	Recorded by seller in study-customized recording form; blood samples collected in both arms	Recorded by seller in study-customized recording form	NA
[35]	Audu	2016	Malaria	NA	NA	Recorded by seller	Recorded by seller	NA
[36]	Aung	2015	Malaria	Mystery client	NA	Household surveys; Mystery client interview	NA	NA
[24]	Awor	2014	Malaria and pneumonia	Direct observation by field supervisor (nurse)	NA	Exit interviews; household survey; direct observation	Exit interviews	NA
[37]	Awor	2015	Malaria and pneumonia	Direct observation by field supervisor (nurse)	NA	Exit interviews; household survey; direct observation	Exit interviews	NA
[38]	Cohen	2012	Malaria	NA	Initial report by WHO/FIND on POCT test, checked every 3 months with 4 unused tests sent for testing	Sales data, administrative records from wholesale distributor, household surveys	Household surveys	NA
[26]	Hansen	2017	Malaria	NA	Microscopy of on-site blood slides	Pharmacy records and surveys	Pharmacy records	Prior willingness to pay study
[39]	Hutchinson	2015	Malaria	No quantitative data	NA	NA	NA	Given for free, can do what they want
[40]	Hutchinson	2017	Malaria	No quantitative data	NA	Information recorded by drug seller and then follow up interviews with questionnaires to clients	Information recorded by drug seller and then follow up interviews with questionnaires to clients (i.e. not based on pharmacy records alone)	Fixed by study authors
[41]	Ikwuobe	2013	Malaria	NA	Batch testing replicating field conditions	Epi-Info version 7 questionnaire	NA	Free
[42]	Kitutu	2017	Malaria, pneumonia and bloody diarrhoea	Direct observation every month	NA	Pharmacist records, exit interviews, direct observation	Pharmacist records, exit interviews, direct observation	NA
[43]	Kwarteng	2019	Malaria	POCT results independently confirmed by lab technician 1 h after results	NA	Participants' questionnaire, focus group discussion, in-depth interview	Participants' questionnaire, focus group discussion, in-depth interview	NA
[44]	Maloney	2017	Malaria	NA	NA	Outlet surveys, customer exit interviews	Customer exit interviews	Willingness to pay responses from pre-intervention exit interviews, comparison w analogous commodities, price negotiation with wholesalers
[34]	Mbonye	2015	Malaria	NA	Used mPOCTs results were routinely checked by research team, confirmed with microscopy testing of blood samples	Vendors recorded data into a specific register	Vendors' data, follow-up interview on 4th day post-visitation	Willingness-to-pay study prior to intervention
[33]	Mboyne	2015						
[45]	Onwunduba	2023	Respiratory tract infections	NA	NA	Mystery clients themseleves recorded data on the visit on structured data collection form	Mystery clients themseleves recorded data on the visit on structured data collection form	RDTs were provided and pharmacies were asked to charge < 1 USD per test
[46]	O' Meara	2016	Malaria	NA	NA	Customized electronic data collection form	Customized electronic data collection form	NA
[47]	Poyer	2018	Malaria	Direct observation by supervisors, mystery client visits	NA	Client exit interviews, mystery client visits	Client exit interviews, mystery client visits	NA
[48]	Shelus	2023	Malaria	NA	NA	Drug shop vendors completed data collection form for each eligible client	Drug shop vendors completed data collection form for each eligible client	NA
[49]	Simmalavong	2017	Malaria	NA	NA	NA	NA	NA
[50]	Soniran	2022	Malaria	Mystery client	NA	Pre and post-intervention household surveys; mystery client surveys	Pre and post-intervention household surveys; mystery client surveys	NA
[51]	Sudhinaraset	2015	Malaria	NA	NA	NA	NA	NA
[52]	Thet	2021	Malaria	Survey of drug shop providers	NA	Survey of drug shop providers	Survey of drug shop providers	NA

Studies collected data on POCT implementation and/or treatment decisions by forms/questionnaires filled out by drug vendors [23, 34, 35, 41, 46, 50, 53], direct observation of the vendors or mystery clients [24, 36–38, 42–45, 47, 52]. Patient-based methods included sales data, administrative/patient records [26, 38] or provider/household surveys [24, 36–38, 44, 50, 52]. Simmalavong et al. (2017) used epidemiological data [49]. Qualitative methods included focus group discussions [39, 40, 43] or interviews with providers [51, 52].

To assess accuracy of POCT administration, studies used mystery clients [23, 36, 45, 47], direct observation [24, 37, 42, 44], microscopy of blood slides [18, 34, 43], or checking POCTs [43]. Two studies reported manufacturer’s specificity and sensitivity [23, 35]. Three assessed sensitivity and specificity directly by comparing malaria POCT results to blood microscopy [26, 34, 41]. Cohen et al. (2012) randomly checked unused tests [38].

Few studies reported how they determined retail price if authors recommended a specific price. Three malaria studies referenced previous willingness-to-pay studies [26, 34, 44].

### Testing and treatment outcomes

In general, studies showed that implementation of POCT could lead to feasibly high uptake levels and adherence to treatment guidelines (Table 3).

**Table 3:** Outcomes of testing and treatment

Ref no	First author	Year published	Sample size	Intended treatment for the Targeted Disease	Description of intervention arm(s)	POCT uptake	POCT positivity (% of patients receiving an POCT who tested positive)	Treatment provision: proportions of all study participants receiving treatment(s) intended for those testing positive
[23]	Ansah	2015	4603 clients attending 24 clusters of shops (each containing 1 to 5 shops)	Artemisinin combination therapy: amodiaquine-artesunate, arthemeter-lumefantrine, or dihydroartemisinine-piperaquine	Shops were trained to carry out a malaria POCT before dispensing medication	100%	49.70%	Based on POCT results: 78.8% (2142/2719, based on POCTs) 65.3% (3005/4603, based on research slides)
[35]	Audu	2016	1200 at 6 private retail pharmacies	Artemether-lumefantrine	Shops were trained on the use of malaria POCT before dispensing medication	NA	43%	Control arm: 98.2% Intervention arm: 78.3% (100% of those of tested positive and 62% of those who tested negative)
[36]	Aung	2015	832 fever cases at 631 POCT outlets	Antimalarials	Arm 1: price subsidy for POCT resupply and monthly check-in visit. Arm 2: price subsidy for POCT and financial/product-related incentives. Arm 3: price subsidy for POCT and monthly intensive support visits by health officers	Arm 1: from 3.0% to 6.4% Arm 2: from 2.7% to 11.9%, Arm 3: from 5.4% to 13.0%	NA	NA
[24]	Awor	2014	Intervention: 487 children with fever at across 44 shops Control: 275 children with fever across 40 shops	Antimalarials for malaria or amoxicillin, oral rehydration solution and zinc sulfte tablets for pneumonia	Shops were trained and provided with subsidised diagnostics and drugs and a community awareness campaign, following “integrated community case management”-style intervention	From 0% to 87.7% of children with fever (427/487)	75% (44/47, based on direct observation)	70.4% (343/487, based on exit interviews) 70.2% (33/47, based on direct observation)
[37]	Awor	2015	6140 children with fever across 44 shops	Antimalarials, oral rehydration solution and zinc sulfte tablets for pneumonia	Shops were trained and provided with subsidised diagnostics and drugs and a community awareness campaign, following “integrated community case management”-style intervention	97.5% of children with fever (5986/6140)	85.1% (5096/5986)	85% (5218/6140)
[38]	Cohen	2012	58 villages offering POCTs (87% of total number of villages)	Artemisinin-based combination therapy or other antimalarials	Shops were trained on tests and told to proceed as they would normally based on whether they thought the client had malaria or not after the test	16% of those with fever	89%	ACT 32% of those testing positive 9% of those testing negative 26.4% of those not tested at all Other antimalarials 66.4% of those testing positive 33.3% of those testing negative 35% of those not tested at all
[26]	Hansen	2017	7522 in intervention, 5797 in control arm	Artemisinin combination therapy	Drug shops trained on how to perform and interpret malaria POCT and prescribe subsidised ACT based on POCT results	100%	43.50%	Intervention: 61% Control: 100%
[39]	Hutchinson	2015	21 focus group discussions (no info on how many people per focus group) 12 months after implementation	Artemisinin combination therapy	Drug shops were trained on POCT usage and decided to treat based on results of POCT			
[40]	Hutchinson	2017	Staff from 59 drug shops, divided into 21 focus groups (each having btw 5 to 13 participants)	Artemisinin combination therapy	Pharmacies were trained to recognise malaria based on POCTs and decide to treat based on the POCT outcome	97.60%	57.50%	52.70%
[41]	Ikwuobe	2013	619 patients in intervention, 607 in contorl, btw 2 pharmacies; total of 1226 participants	Antimalarials	Pharmacists were trained on and provided with POCTs and tested those with anti-malarial prescription or wanting to self-medicate with them, then allowed to proceed with dispensing of drugs following discussion between pharmacist and patient	NA	13.6% (84/619)	58.2% (360/619)
[42]	Kitutu	2017	3738 child fever cases across 61 drug shops in intervention arm	Artemisinin for malaria, amoxicilllin for pneumonia, zinc sulfate solution for non-bloody diarrhoea	Drug shops were trained in integrated community case management to provide POCT testing for children with malaria, consisting of training of drug sellers, provision of information, education and communication, supplying diagnostics and medicines, and monthly supportive supervision	97% (3628/3738)	47% (1957/4190)	NA
[43]	Kwarteng	2019	1973 clients across 42 licensed chemical shops	Antimalarials	Pharmacies and licensed chemical shops were trained on use of malaria POCTs and treated based on national malaria treatment guidelines	NA	60.2% (1081/1797)	60.2% (1082/1797)
[44]	Maloney	2017	1214 patients across 262 drug dispensing outlets	Antimalarials	Arm 1: training, access to and supervision on use of POCTs to treat clients Arm 2: same as Arm 1 but also received subsidised POCTs and sold at subsidized price	Intervention: increased from 19 to 74%, cntrol: increased from 3 to 18%	41%	NA
[34]	Mbonye	2015	15517 patients (8672 intervention and 6845 control) across 59 drug shops	Artemisinin combination therapy	Drug shops were additionally trained on use of malaria POCT, and asked to manage patients based on POCT results using subsidized ACT	97.8	58.50%	Intervention: 62.5% Control: 99.8%
[33]	Mboyne	2015	Policy implications of Mboyne et al. (2015)					
[45]	Onwunduba	2023						
[46]	O' Meara	2016	444 participants across 11 shops	Artemisinin combination therapy	Arm 1: Free POCT and conditional ACT subsidy Arm 2: Free POCT but no ACT subsidy Arm 3: No POCT subsidy but conditional ACT subsidy Arm 4: No POCT or ACT subsidy	Arm 1: 73.7% Arm 2: 73.8% Arm 3: 49.6% Arm 4: 51.0%	Arm 1: 39.3% Arm 2: 27.6% Arm 3: 44.6% Arm 4: 47.1%	Arm 1: 43.9% Arm 2: 25.9% Arm 3: 32.8% Arm 4: 29%
[47]	Poyer	2018	633 clients in second round at 120 outlets, but high rates of dropout	Antimalarials	Private health facilities (PHF) and pharmacies (P) were trained in use of malaria POCTs and offered them to febrile patients	PHF: from 30.4% to 52.6% P: from 52.1% to 56.3%	PHF: from 52.3% to 45.1% P: from 47.2% to 52.8%	PHF; from 42.5% to 41.8% P: from 31% to 29.4%
[48]	Shelus	2023	934 clients of drug shops	Antimalarial	Drug shops offered malarial POCTs to febrile clients or clients seeking antimalarials for themselves or for others and recorded the medication purchased by these clients	36%	43%	79.40%
[49]	Simmalavong	2017	2,301,676 tests across 317 pharmacies	Artemether/lumefantrine	Private pharmacies were trained on and supplied with POCTs and antimalarials to diagnose and treat malaria as part of a public–private scheme to increase diagnostics in private clinics, which was then scaled up over time	NA		
[50]	Soniran	2022	637 caregivers of febrile children under 10 yo and 48 mystery cleint visits	ACT	Over-the-counter medicine selllers were trained to sight patients suspected of malaria and conduct a test on thembefore prescribing antimalarials to patients testing positive	30.8% (intervention; household survey) or 38.1% (intervention; mystery client); 10.5% (control; household survey) or 23.3% (control; mystery client)	25.0% (intervention, mystery client) vs 42.9% (control, mystery client)	33.3% (intervention, mystery client) vs 53.3% (control, mystery client)
[51]	Sudhinaraset	2015	30 informal providers of POCTs	Antimalarials	Arm 1: subsidised POCTs Arm 2: subsidised POCTs and free POCT for every 5 purchased by providers Arm 3: subsidised POCTs and information, education and counselling	NA		
[52]	Thet	2021	80 malaria drug shops for quantitative and 65 of these for qualitative interviews	"Effective and quality" antimalarial drugs	Drug shops participated in nationwide project to replace widespread use of artemisinin monotherapy with combination therapy, and were trained to perform malaria POCT to guide management of clients	NA	NA	NA

Ref no	Treatment provision: % of patients not tested receiving the intended treatment for the study's targeted diseases	Adherence: % of patients with a negative POCT result not receiving the intended treatment for the study's targeted diseases	Adherence: % of patients with a positive POCT result receiving the intended treatment for the study's targeted diseases	% of patients tested positive referred elsewhere by the provider for further care	Safety & Accuracy of administration of testing (% of providers who could accurately perform an POCT, read its result and dispose of waste)	Accuracy of POCT (sensitivity, specificity, positive predictive value or other quality measures)	Retail price (USD)
[23]	Control: 93% (slide-positive), 88% (slide-negative)	97%	99.50%	0.52% (7/1351)	87.2% to 100% of different safety indicators	Sensitivity: 98–100% by shop Specificity: 73% to 98%, some 30%, 31%, 52%	0
[35]	98.20%	38%	100%	NA	NA	Sensitivity: 97.3% Specificity: 98.5% Positive predictive value: 98.0%	NA
[36]	NA	NA	NA	NA	94%	NA	0.18
[24]	NA	90.9% (10/11, based on direct observation)	100% (33/33, based on direct observation)	NA	94%	NA	0
[37]	NA	NA	93.5% (4961/5307)	NA	NA	NA	NA
[38]	61.40%	57.70%	98.40%	NA	> 95% for POCT administration and procedure adherence	Lot-testing: 100% passed	0.40 (ranged between 0–2)
[26]	100%	98.60%	99.10%	NA	NA	Sensitivity: 91.75% Specificity: 62.92%	0.2
[39]							
[40]	99.20%	70.30%	94.30%	NA	NA		2 (between 0.08–13.20 USD in mPOCT negative); 1.62 (between 0.12–12.80 USD in mPOCT positive); 1.32 (between 0.32–18.00 USD in control arm)
[41]	Control: 100% (607/607)	48.4% (259/535)	100% (84/84)	NA	NA	Sensitivity: 100% Specificity: 100%	0
[42]	NA	NA	73.9% (based on exit interviews) 88.7% (based on pharmacist record) 63.3% (based on direct observation)	NA	NA	NA	0
[43]	NA	73.9% (529/716)	82.79% (895/1081)	NA	94.9% (1873/1973)	NA	NA
[44]	Intervention: 35% Control: 41%l	93%	90%	NA	NA	NA	0.32 (subsidised) and 0.67 (not subsidised)
[34]	Intervention: 51.4%-73.7% Control: 99.6%-100.0%	98.5% (3117/3166)	99% (4858/4907)	NA	95% of POCTs read correctly	Sensitivity: 91.7% Specificity: 63.1%	0.2
[33]							
[45]							
[46]	Arm 1: 30%, Arm 2: 29.6%, Arm 3: 21.1%, Arm 4: 26.5%	Arm 1: 72.5%, Arm 2: 80.0%, Arm 3: 87.1%, Arm 4: 92.6%	Arm 1: 81.8%, Arm 2: 71.4%, Arm 3: 84%, Arm 4: 58.3%	NA	NA	NA	0 or 0.50
[47]	PHF: from 8.4% to 19.8% P: from 40.8% to 22.2%	PHF: from 93.3% to 96.1% P: from 86.1% to 100%	PHF: from 84.6% to 91.6% P: from 86.8% to 92.6%	0%	PHF: from 25.3–97.8% to 14.7–100% P: from 33.3–100% to 20.7–97.7%		0.8 or 1 for hospital or single pack POCT respectively (suggested prices)
[48]	87.30%	63.70%	93.60%	NA	NA	NA	0.57
[49]							
[50]	From 47.1% to 66.7% (before and after intervention, household survey) 34.6% (Intervention, mystery client) vs 52.2% (Control, mystery client)	83.3% (Intervention, mystery client) vs 75.0% (Control, mystery client)	75.0% (Intervention, mystery client) vs 100% (Control, mystery client)	NA	66.7% (intervention, mystery client) vs 40% (control, mystery client)	NA	0.44
[51]							
[52]	NA	73.90% of drug shops	NA	NA	NA	NA	NA

This was dependent upon factors like adequately designed training, demand generation, linkage to care, support for providers, and appropriate financial remuneration. For example, in one longitudinal study of malaria POCTs, uptake increased when monthly check-ins, financial incentives, or more intensive support was implemented in subsidized schemes of POCTs, from 3.0% to 6.4% (monthly check-ins), 2.7% to 11.9% (financial incentives), and 5.4% to 13.0% (intensive support) [36]. Moreover, six studies reported adherence of treatment outcome to positive and negative test results above 90% [23, 24, 26, 34, 44, 47]. These factors will be discussed individually below.

#### Training for providers

POCT implementation requires comprehensive training before implementation and our review finds that it should cover topics including POCTs’ importance and benefits, its administration, interpretation, waste disposal, and counselling after results [40, 54, 55]. Firstly, emphasising the need, value, and accuracy of POCTs may improve uptake. In a Kenyan malaria study that only taught epidemiology and POCT procedure/management but did not emphasise the need for POCTs, uptake was as low as 30.4% [47]. Conversely, when providers were educated on POCT’s value and felt aligned with professionals through training, higher provider uptake was observed [36, 51, 56, 57], reaching 97% in a study where drug shops were trained in an integrated community case management style where providers were educated on the need and how malaria POCTs worked [42]. Instilling belief in the need for POCTs could address other factors limiting POCT uptake – for example, some providers only used POCTs for specific patient profiles or disobeyed guidelines, believing NGOs had eradicated local malaria [51] or endemicity was declining [52]. Alternatively, some providers relied on clinical judgement if they perceived shortcomings of POCTs [35]. One study found poor adherence to negative malaria POCT results, as 20–41% of malaria-negative mystery clients were told by providers that they were positive and suggested that this was due to providers’ “mistrust of POCT results” despite their competence in POCT performance [47]. This is despite high sensitivity of malaria POCTs (91.7%–100% [23, 34]), which could be highlighted in training to instil confidence.

Secondly, training must include clear guidelines for negative tests. Studies with the worst adherence to negative test results – 48.4% in Ikwuobe et al. (2013) and 57.7% in Cohen et al. (2012), which also had the poorest uptake (16%) – had (sometimes deliberately) vague guidelines [38, 41]. The former lacked guidelines on handling negative results and suspension of antimalarials only after a pharmacist/patient discussion without study authors [41]. In the latter, staff were to proceed as “[they] would normally”, only being told “how POCTs work and how to use them” [38]. Hutchinson et al. (2017) reported vendors’ “anxiety around the management of a negative mPOCT [malaria POCT] result” as negative results might reveal “diagnostic uncertainty” regarding the illness, negatively impacting vendors’ reputation [40]. Hence, managing negative test results well is important for provider uptake and to reassure customers. This is also important from the providers’ perspective to maintain drug sales, which will be discussed below.

Guidelines for negative tests appear to be particularly important for diseases in which concerns about under prescription are dominant. Of studies with large discrepancies between adherence to positive versus negative test results, studies tended to report higher adherence to positive results than negative (Table 4) [35, 38, 40, 41, 53]. This could reflect a reluctance to under-prescribe medication and miss a diagnosis in malaria. In contrast, in other studies of notifiable diseases like HIV and HCV, low positive adherence may indicate stigma: for example, an HCV study in a high-income country (excluded from this review) reported 100% adherence to negative test results but only 28.2% adherence to positive test results [58]. However, data on this in the studies included in this review is limited, as the single study on CRP POCTs only reported adherence to negative CRP test results (30.4%) as they only collected data through mystery clients [45].

**Table 4:** Studies where positive and negative adherence differ

Study	Disease	Positive adherence (%)	Negative adherence (%)
Audu et al. 2016 [35]	Malaria	100	38
Cohen et al. 2012 [38]		98.4	57.7
Hutchinson et al. 2017 [40]		94.3	70.3
Ikwuobe et al. 2013 [32]		100	48.4
Shelus et al. 2022 [59]		93.6	63.7

Thirdly, providers should consider the format in which training is implemented. Certification of a formal training course could assure customers of providers’ credibility and instil self-confidence in providers, particularly in LMICs [26, 34, 39, 40]. For example, Klepser et al. mandated a “Collaborative Institutional Training Initiative program” and POCT certificate course [60–62]. This would capitalize upon the increased legitimacy in the eyes of customers already conferred upon vendors using POCTs. For example, customers were surveyed on their opinion of the outlets administering POCTs. In Uganda, POCTs gave legitimacy to vendors, who were perceived to have unclear credentials [33, 39] or purely profit-driven [40]. This change was attributed to the involvement of external project supervisors/government and new technology [39], with sharps and blood testing shifting vendors into the “category of an endorsed professional” [34, 40]. Such outward-projecting improvements in retailers’ image also benefited vendors: for example, in Uganda, vendors reported outward-directed benefits, as government partnership conferred legitimacy, status, and confidence about safety from authorities’ raids [39]. They attributed this to POCT technology marking them as endorsed professionals, particularly visibly drawing blood using recognisably medicalized objects like gloves, needles, and packaged lancets [39]. Hence, formalization of training and certification in POCT could reinforce these benefits and improve uptake amongst customers by legitimizing shops. Studies performed after the COVID-19 pandemic, in which pharmacists took on a big role in testing, might show further changes in this direction of customers’ attitudes toward pharmacist-performed POCT, as has been shown for high-income countries [63–66]. However, none of the studies performed after the pandemic directly assessed changes in these attitudes.

Some customers worried sellers were “unskilled” in the practice of POCTs and risked HIV infection [51] or injury, or overcharged [40]. In contrast to these concerns, six studies reported that POCTs were accurately performed, with performance of measures of safety/test administration/waste disposal above 90% [23, 24, 34, 36, 38, 43] and vendors felt more confident about making medical decisions by reducing guesswork [40, 51, 67]. However, a few studies like Poyer et al. (2018) reported more inconsistent levels of accuracy, between 14.7% and 100% attainment of safety outcomes [47], while Soniran et al., (2022) reported 66.7% attainment of safety outcomes compared to 40% in control arms [50]. Some commonly missed steps before the procedure included not checking expiry dates [38], explaining the test, or testing away from other clients [47]. Steps missed during the test were drawing the right amount of blood [38, 50], using antiseptic [36], wearing gloves [47], checking the time after adding the buffer [50]. Afterwards, providers failed to immediately dispose of lancets in sharps bins [47, 50], or waited < 15 min before reading the result [47]. We recommend that a checklist be provided, highlighting these commonly missed steps to ensure that the proper procedures are followed.

#### Demand generation and community sensitization

Community sensitization can help potential customers recognise the importance of POCT and appropriate treatment [24, 54, 57, 68]. Patients were more likely to consent to receiving a POCT if they had used one before or were aware of its availability [44]. For example, two studies in this review with the highest adherence to test results implemented a POCT program that integrated with a community awareness campaign [24, 37]. Conversely, lack of familiarity contributed poor uptake in some studies which lacked any demand generation [27, 69–71], as evidenced in studies like Cohen et al. (2012), and Ikwuobe et al. (2013). Community sensitization could improve customers’ acceptance of test outcomes and reduce pressure on providers to meet customers’ expectations, improving provider adherence too [47, 51]. Furthermore, some providers did not comply with national malaria guidelines as they could not ensure patients finished the full antimalarial course [52] – community sensitization could teach patients to receive treatment properly.

The content of sensitization should emphasize the benefits of testing at private retailers without diminishing other healthcare sectors: for example, a qualitative study suggested that sensitization efforts should focus on emphasising the need for testing regardless of the location (public or private), and leverage the trust in drug shops, which could increase uptake at private retailers without diminishing the work of public healthcare [59]. In the reviewed studies, over 80% of *customers* were satisfied with POCTs [34, 72–74], citing the convenience of testing locations [51, 74], as they would not have to travel to hospitals [40].

Programs need to consider the most effective methods of community sensitization, which may vary depending on the level of economic development or degree of trust in medical technology in the area. For example, two studies communicated the need for POCTs through community leaders, which may be better trusted by communities in LMICs [23, 50]. None of the included studies surveyed the participants on where they had heard of the POCT service, a question that could be included in future questionnaires to evaluate the most effective form of advertising.

#### Linkage to care

Several studies identified formal linkage to support as important for success. Barriers included resistance from healthcare workers to the POCT program [34] and failure of patients to honour referrals [43]. For example, health-workers were concerned about the same issues that were reported by private sellers as being beneficial to private retailers. They felt untrained vendors encroached on professional boundaries, deeming them untrustworthy, and did not acknowledge the paperwork vendors used to refer customers [39]. Health-workers also worried about decreased malaria testing at their facility [39]. However, some health-workers recognised improvements like customers’ shorter travel times [40].

Suggestions to bridge public and private sectors include capacity-building programs for pharmacists with health workers to address mistrust of health workers [43] or partnership with government agencies and professional bodies [47]. Community sensitization on accreditation of pharmacists and the formalisation of training could address health workers’ preconceptions about unprofessionalism [39]. Secure communication platforms should be established to protect data security. Two studies further proposed integrating POCT programs into nationwide malaria surveillance systems [44, 49].

#### Support for providers

Adequate provider supervision and support is important immediately after POCT implementation and in the long run. One study found price subsidies for providers to buy POCTs were most effective when accompanied by monthly intensive support as it led to a larger increase in uptake (from 5.4% to 13%) than just check-in visits (from 3% to 6.4%) [36]. Another study commented that prolonged support is necessary because provider behaviours, particularly for malaria, are “driven by ingrained behaviours” and thus difficult to change quickly [23]. This was echoed in focus group discussions with providers, as although some providers felt POCTs were easy to use and optimised workflow by reducing guesswork [51], others felt their workload had increased. However, adequate support and minimizing the Hawthorne effect need to be balanced [34]. The Hawthorne Effect can occur when practitioners modify their behaviour knowing that they are being monitored during interventional studies [34]. A possible support schedule could provide intensive support initially and decrease intensity over time [75, 76].

In LMICs, as well as individual provider support, other forms of support that should be considered in POCT implementation programs include addressing systems-level barriers [51, 77]. Providers relied on research teams for waste disposal or struggled to collaborate with public health facilities [51, 77]. Hence, this study recommends that a systematic approach to integrating private retailers into waste management systems and infrastructure be considered for safe POCT disposal.

Other systemic factors relating to procurement included lacking weighing scales to calculate antimalarial dosage [43], or being undeliverable due to flooded roadways [44]. Simmavalong et al. (2017) described private facilities’ lack of control over POCT distribution left them vulnerable to “trickle down shortages” in their government-led program [49]. In one study, some shops disobeyed malaria guidelines as they lacked POCTs (3/65) or antimalarials (1) despite free central provision [52]. When designing POCT programmes in LMICs, comprehensive material and logistical support in addition to the POCTs themselves needs to be included.

#### Financial remuneration and pricing

POCT pricing should be carefully evaluated through willingness-to-pay studies, as it impacts provider income and patient uptake. Factors like manufacturing subsidy, distribution, cost of training, equipment, or supervision should be balanced against the price customers are willing to pay. Especially in LMICs, where the “combined cost of mPOCT [malaria POCT] and ACT is a barrier to rural folks” [43], a subsidy may be necessary to facilitate patient uptake.

Seven studies offered POCT for free to customers [23, 24, 35, 37, 41, 43, 46], while seven supplied POCT for free but sold at a subsidized price [26, 34, 38, 40, 44, 46, 47, 50], and the remaining studies did not explicitly state the price at which they were sold [35, 43, 49, 51, 52]. In Hutchinson et al. (2015), providers were given tests for free and sold at providers’ chosen price [39]. Retail prices for customers ranged between 0.18 USD (Myanmar [36]) to 2 USD (Uganda [40]).

These different financial schemes have been evaluated to mixed results – in one malaria study, there was no difference in uptake between districts that were or were not subsidised for POCTs [44]. Similarly, providing another financial incentive in addition to price subsidies did not significantly increase uptake compared to price subsidy with intensive support [36]. In contrast, a third study reported malaria POCT subsidies positively impacted uptake independent of a prior offer of an antimalarial subsidy, suggesting that subsidies influence the “next immediate action” (deciding whether to test) [46]. A cost-effectiveness analysis of malaria POCT in Myanmar also found that price subsidy coupled with information, education and communication provider-targeted strategy was the most cost-effective [78]. Possible cost-saving measures included reducing supervision, having shops cover some training fees or gloves.

Providers’ attitudes towards renumeration may be affected by disease endemicity and negative test management. For illnesses like malaria where negative tests could impact drug sales directly, there was “tension between the motivation of the shop owner to make a profitable drug sale” and the lack of income after a negative test [46]. This was echoed in Gwagwalada, Nigeria, considered meso-endemic for malaria, where reduced antimalarial purchase may generate significant income loss [41]. A pharmacist “expressed concerns about loss of sales”, reasoning it would be difficult to restrict profit-guided pharmacies’ antimalarial sales without alternative income [41].

In studies with high malaria positivity, providers felt the programme benefited from them financially, as they enjoyed increased drugs and POCT sales [24, 39]. Even negative tests offered opportunity to sell more drugs, changing from antimalarials to other drugs like antipyretics [23, 26], and greater polypharmacy and median spending by those testing negative [40]. However, drugs like paracetamol have a “lower profit margin”, so whether overall economic benefit occurs may be affected by the proportion of positive/negative tests in high/low endemic areas [37]. These further highlight the importance of providing clear guidelines for negative test results, as described earlier.

### Quality assessment

Study quality varied greatly (Table 5): only 11 studies had a randomised research design and a control group, of which seven corrected for confounding factors. Although 17 studies were multi-centre, sample sizes varied drastically: some were extremely small with only 21 focus groups [39], while others reached 15,517 patients across 59 drug shops [34]. An epidemiological study pooled 2,301,676 tests across 317 pharmacies [49]. Three studies did not disclose funding sources [36, 41, 51] (Additional file 3).

**Table 5:** Quality assessment of the included studies

Ref no	Author	Published year	Target Disease	Randomized research design used	Availability of external control group	Multiple single centre?	Sustainability of the intervention sufficiently assessed (> 12 months)	Sample size calculation (except for qualitative studies)	Prospective data collection	Correction for confounding factors	Total no. of criteria met (out of 7)
[23]	Ansah	2015	Malaria								0
[35]	Audu	2016	Malaria								0
[36]	Aung	2015	Malaria							✓	1
[24]	Awor	2014	Malaria and pneumonia			✓		✓			2
[37]	Awor	2015	Malaria and pneumonia			✓			✓		2
[38]	Cohen	2012	Malaria			✓	✓				2
[26]	Hansen	2017	Malaria	✓	✓	✓	✓	✓	✓	✓	7
[39]	Hutchinson	2015	Malaria	✓	✓	✓	✓	Qualitative	✓		5
[40]	Hutchinson	2017	Malaria	✓	✓	✓	✓	Qualitative	✓		5
[41]	Ikwuobe	2013	Malaria	✓	✓			✓	✓	✓	5
[42]	Kitutu	2017	Malaria, pneumonia and bloody diarrhoea		✓	✓	✓	✓	✓	✓	6
[43]	Kwarteng	2019	Malaria			✓		✓	✓		3
[44]	Maloney	2017	Malaria	✓	✓	✓	✓	✓	✓	✓	7
[34]	Mboyne	2015	Malaria	✓	✓	✓	✓	✓	✓	✓	7
[46]	O' Meara	2016		✓	✓	✓		✓	✓	✓	6
[47]	Poyer	2018	Respiratory tract infections			✓	✓	✓	✓		4
[49]	Simmalavong	2017	Malaria			✓	✓		✓		3
[51]	Sudhinaraset	2015	Malaria	✓	✓						2
[52]	Thet	2021	Malaria	✓		✓	✓				3
[45]	Onwunduba	2022	Malaria	✓		✓			✓		3
[50]	Soniran	2022	Malaria	✓	✓	✓	✓	✓	✓		6
[48]	Shelus	2023	Malaria			✓			✓		2

Moreover, differences in methods of measurement between studies hampered direct comparison – one study measured uptake as proportion of households remembering receiving POCT [36] while others only included consenting clients [23, 34]. In one study, there was significant heterogeneity in willingness to provide testing amongst 92 drug shops [38]. A standardised way of quantifying POCT uptake should be used across studies to allow for comparison – for example, recording the proportion of febrile clients or clients suspected of a particular disease by the pharmacist.

## Discussion

In this review, we describe that many studies demonstrated POCT can improve diagnosis, referral, and treatment of infectious diseases [23, 24, 26, 34, 37, 44, 47]. Pharmacies are a potential point of intervention to manage infectious disease diagnosis and treatment/referral [48]. In Uganda, > 72% of care-seekers sought care for febrile children at drug shops [42, 64]. Pharmacies increase accessibility in terms of proximity to patients’ houses, cost, and opening hours compared to primary care [64]. In one study, 38% of patients presented outside normal clinic hours [62]. This may benefit populations in LMICs, whose inconsistent schedules, language barriers, or unreliable internet complicate appointment-scheduling [79]. Other studies echo the importance of pharmacies for non-emergency care-seeking [23, 80–82].

### Comparison with literature

This review adds to a small body of literature on POCT in private retail settings [83]. No other review examined infectious disease testing, except Visser et al. (2016) on malaria POCTs [77]. Of the twelve papers studied, five were absent from our review: four were unpublished, while the published study compared three training intensities and sensitization and reported little effect on an overall poor uptake [84]. Although study authors reported uptake and adherence improved with longer training, frequent initial supervision, and low POCT prices, this was only based on three studies with lowest provider numbers, with multiple exceptions [77]. The authors also argued that POCT programs would not scale-up easily, but that less intense but more scalable programs had poorer outcomes [77]. This was more difficult to compare in our review considering our greater number and heterogeneity in studies. However, the authors cited similar concerns like waste management, provider and client expectations, training, and wider health-system integration [77].

Another review by Boyce et al. (2017) on malaria POCTs in sub-Saharan Africa across private, public and community healthcare included five retail studies also assessed in our review, and agreed that POCT providers had good adherence, execution, and sensitivity, but lacked specificity compared to community health workers and formal healthcare [85]. Their other concerns mirrored ours, adding that patients may purchase substandard non-ACT antimalarials, contraindicating the intent of POCTs [85]. Nevertheless, the authors’ comparison with other healthcare settings highlights the usefulness of retail settings [85].

### Strengths and limitations

Our search strategy with two independent researchers means it is unlikely we missed many eligible studies within PubMed/Medline. However, five studies were neither randomised nor controlled, four were quasi-experimental (in which participants are assigned to control and intervention groups in a non-random manner), and another four were pilot studies that lacked a control arm for comparison. Moreover, under close supervision, retailers may behave differently than when unwatched – the Hawthorne Effect [34] – highlighting the usefulness of evaluation via mystery clients. Initial implementation schemes could consider a period of evaluation using this method to assess the quality of POCT performance. Moreover, data collection relying on self-reporting like household surveys/exit interviews or pharmacist questionnaires are subject to bias/recall issues. For example, Cohen et al. (2012) reported differences between POCT positivity reported by customers and providers (89% versus 60%), as customers may not admit to buying antimalarials after testing negative [38]. Recall bias may be prominent after long follow-up periods [28].

This review is limited to English studies on PubMed/Medline. High heterogeneity prevented formal meta-analysis. Most studies were on malaria, especially qualitative discussions, so outcomes are biased towards malaria studies. This review highlights the need for quality primary POCT research, particularly for non-malaria infectious diseases. For instance, there is little data on CRP, other than to differentiate viral and bacterial infections in primary healthcare [86].

## Conclusion

Private retail providers are an important point-of-access for patients, facilitating prompt diagnosis and treatment. In LMICs, POCT interventions can improve treatment-seeking behaviour, reduce inappropriate antimicrobial use and resistance, and lessen the burden on public healthcare services. This review shows POCT is not only feasible in non-formal settings but also welcomed by shops and customers. Successful implementation in LMICs requires a comprehensive protocol, including community sensitization, training, reasonable pricing, infrastructure support for low-resourced pharmacies, and wider healthcare integration.

### Supplementary Information

**Additional file 1.** Full search terms.

**Additional file 2.** Abstract screening tool.

**Additional file 3.** Table of funding.

## Data Availability

All primary research articles are publicly available and searchable on PubMed.

## Abbreviations

POCT: Point-of-care testing
AMR: Antimicrobial resistance
AMS: Antimicrobial stewardship
LMIC: Low- and middle-income countries
CRP: C-reactive protein
RTI: Respiratory tract infection
mPOCT: Malaria point-of-care test
HCV: Hepatitis C virus
