# Supplementary material for: Point-of-care testing in private pharmacy and drug retail settings: a narrative review
Source: BMC Infect Dis. 2023 Aug 23;23:551. doi: 10.1186/s12879-023-08480-w (PMC10463283; doi:10.1186/s12879-023-08480-w)
Supplement: Supplementary file 1 — Additional file 1. Full search terms. [file 12879_2023_8480_MOESM1_ESM.docx]

Additional file 1: full search terms (Additional file 1.docx)

| **Population (infectious disease)** | **Intervention (rapid diagnostic testing)** | **Outcome (pharmacy)** |
| --- | --- | --- |
| Communicable diseases | Self-test | Pharmacies |
| Infectious diseases | Rapid diagnostic test | Pharmacy |
| Bacterial infections and mycoses | Point-of-care testing | Community pharmacy service |
| Respiratory tract infections | RDT(s) | Drug shop |
| Virus diseases | POC(s) | Medicine retailer |
| influenza | Diagnostic tests | Drug vendor |
| Group A streptococcus | Routine/methods | Private sector |
| Hepatitis C | Reagent kits | Commerce |
| HCV | Diagnostics | Commercial sector |
| malaria | Point-of-care test | Retail sector |
| Parasitic diseases |  | Private provider |
| HIV |  | Private outlet |
| Sars-CoV-2 |  | Medicine shop |
|  |  | Informal providers |
|  |  | Patent medicine vendor |
|  |  | Over-the-counter |
|  |  | unregulated |
|  |  | profit |

((((((((((((((((((communicable disease) OR (infectious disease)) OR (bacterial infections)) OR (respiratory tract infections)) OR (viral disease)) OR (influenza)) OR (Group A streptococcus)) OR (Hepatitis C)) OR (HCV)) OR (malaria)) OR (Parasitic disease)) OR (HIV)) OR (Sars-CoV-2)) AND (((((((((((((((((((((((("self-test") OR ("self-tests")) OR ("self-testing")) OR ("rapid diagnostic test")) OR ("rapid diagnostic testing")) OR ("rapid diagnostic tests")) OR ("point-of-care test")) OR ("point-of-care tests")) OR ("point-of-care testing")) OR ("RDT")) OR ("RDTs")) OR ("POC")) OR ("POCT")) OR ("POCTs")) OR ("diagnostic test")) OR ("diagnostic tests")) OR ("antigen rapid test")) OR ("antigen rapid tests")) OR ("home test kit")) OR ("home test kits")) OR ("diagnostic routine")) OR ("diagnostic method")) OR ("reagent kit")) OR ("reagent kits"))) AND (((((((((((((((((((((((((((("pharmacies") OR ("pharmacy")) OR ("community pharmacy service")) OR ("drug shop")) OR ("drug shops")) OR ("medicine retailer")) OR ("medicine retailers")) OR ("drug retailer")) OR ("drug retailers")) OR ("drug vendor")) OR ("drug vendors")) OR ("medicine vendor")) OR ("medicine vendors")) OR ("private sector")) OR ("commercial sector")) OR ("retail sector")) OR ("private provider")) OR ("private providers")) OR ("private outlet")) OR ("private outlets")) OR ("private medicine outlets")) OR ("medicine shop")) OR ("medicine shops")) OR ("informal provider")) OR ("informal providers")) OR ("patent medicine vendor")) OR ("patent medicine vendors")) OR ("over-the-counter"))) NOT (animals (mh) NOT human (mh))) NOT (Review[Publication type])) NOT (meta-analysis[publication type])) NOT (systematic review[publication type])
