# Supplementary material for: Point-of-care testing in private pharmacy and drug retail settings: a narrative review
Source: BMC Infect Dis. 2023 Aug 23;23:551. doi: 10.1186/s12879-023-08480-w (PMC10463283; doi:10.1186/s12879-023-08480-w)
Supplement: Supplementary file 2 — Additional file 2. Abstract screening tool. [file 12879_2023_8480_MOESM2_ESM.docx]

Additional file 2: Abstract screening tool (Additional file 2.docx)

**Citation, Title, and Abstract Screening**

1. Does the title or abstract NOT indicate that an POCT systematic review or meta-analysis was conducted?

a. Yes: continue screening

b. No: stop screening

2. Does the title or abstract indicate that this is NOT a correction or erratum?

a. Yes: continue screening

b. No: stop screening

**Abstract Screening**

3. Does the abstract indicate that a POCT was implemented?

a. Yes or Unsure/Unclear: continue screening

-For example: the study implements POCT with or without supporting interventions

-Key words: self-test, rapid diagnostic test, point-of-care testing, POCT(s), POC(s), diagnostic tests, routine/methods, reagent kits, diagnostics, point-of-care test

b. No: stop screening

-For example: only reporting test accuracy, hypothetical or modelling studies, focus group discussions on POCT without actual implementation

4. Does the abstract indicate that the private sector was studied?

a. Yes or Unsure/Unclear: continue screening

-Key words: pharmacies, pharmacy, community pharmacy service, drug shop, medicine retailer, drug vendor, private sector, commerce, commercial sector, retail sector, private provider, private outlet, medicine shop, informal providers, patent medicine vendor, over-the-counter,

UPDATE using new search terms

b. No: stop screening

-Other settings, not eligible: primary care departments, outpatient clinics

5. Does the abstract indicate that the POCT(s) was/were performed by pharmacy staff?

a. Yes or Unsure/Unclear: continue screening

-The tests were *directly* performed by pharmacy staff

b. No: stop screening

-For example: did not use their staff to perform the POCT

6. Does the abstract indicate that the POCT was used for infectious disease detection?

a. Yes: continue screening

-Key words: communicable diseases, infectious diseases, bacterial infections, respiratory tract infections, virus diseases, influenza, group A streptococcus, hepatitis C, HCV, malaria, parasitic diseases, HIV, COVID-19, coronavirus

b. No: stop screening

7. Does the abstract indicate that the study uses either or both a quantitative and qualitative design to measure feasibility and impact of implementing POCT?

a. Yes or Unsure/Unclear: continue screening

-Key words: POCT uptake, perceptions of providers and customers, adherence to test results, recommended retail price, safety protocols

b. No: stop screening

8. Decision: Should this article be included?

a. Yes, all 9 screening questions answered Yes or Unclear

b. No, at least one answers definitely “No”
