# Supplementary material for: Point-of-care testing in private pharmacy and drug retail settings: a narrative review
Source: BMC Infect Dis. 2023 Aug 23;23:551. doi: 10.1186/s12879-023-08480-w (PMC10463283; doi:10.1186/s12879-023-08480-w)
Supplement: Supplementary file 3 — Additional file 3. Table of funding. [file 12879_2023_8480_MOESM3_ESM.docx]

Additional file 3: Table of funding (Additional file 3.docx)

| **Ref no.** | **Author** | **Funding** |
| --- | --- | --- |
| 23 | Ansah | Malaria Capacity Development Consortium of the London School of Hygiene & Tropical Medicine with funding from the Welcome Trust and the Bill and Melinda Gates Foundation. |
| 52 | Audu | No financial support or sponsorship |
| 39 | Aung | Funding Not disclosed |
| 24 | Awor | Einhorn Family Foundation – Sweden, Medicines for Malaria Venture |
| 40 | Awor | Einhorn Family Foundation – Sweden, Medicines for Malaria Venture |
| 41 | Cohen | Clinton Health Access Initiative and the Bill and Melinda Gates Foundatio |
| 26 | Collins | Secretary’s Minority AIDS Initiative Fund and led by CDC (grant #5U62PS003949 |
| 50 | Czarniak | J M O’Hara Research Fund managed by the Pharmaceutical Society of Western Australia. |
| 51 | Demore | ARS Grand-Est; the Union régionale des professionnels de santé Pharmaciens Lorraine; and ANTIBIOLOR network. |
| 35 | Dong | San Francisco Department of Public Health, Hepatitis Division |
| 48 | Gauld | Green Cross Health, the Pharmaceutical Society and the Pharmacy Guild of New Zealand  honorariums from pharmaceutical companies for speaking on HCV  Chair of the Ministry of Health Hepatitis C Implementation Committee, and Chair of the Northern Regional DHB Alliance Hepatitis C Steering Group |
| 42 | Hansen | ACT Consortium, funded through a grant from the Bill & Melinda Gates Foundation to the London School of Hygiene and Tropical Medicine [grant number 39640] |
| 43 | Hardin | No external funding |
| 34 | Hohmeier | Genentech, Inc  Michael Klepser receives royalties from the National Association of State Pharmacy Associations for a pharmacy-based point-of-care training program. He also has received grant funding from Janssen and conducts market consultation for Qorvo, Inc. Donald G. Klepser receives royalties from the National Association of State Pharmacy Associations for a pharmacy-based point-of-care training program. He also conducts market consultation for Roche Diagnostics, Quidel, Diasorin, and Qorvo, Inc. He serves as a clinical advisor board member and receives honoraria from FDS/Amplicare. Kenneth C. Hohmeier conducts market consultation for Qorvo, Inc. He serves as a clinical advisor board member and receives honoraria from FDS/ Amplicare |
| 33 | Hutchinson | ACT Consortium, which is funded through a grant from the Bill &Melinda Gates Foundation to the London School of Hygiene & Tropical Medicine. Siân Clarke issupported by the Wellcome Trust through a Research Career Development Fellowship (084933) |
| 47 | Hutchinson | Wellcome Trust (084933 (career development fellowship (SEC)); Institutional Strategic Support Fund (CIRC) |
| 44 | Ikwuobe | Not disclosed |
| 49 | Kawachi | JSPS KAKENHI (Grant No. 16 K08431) and a Miyagin Economic Research Institute Grant-in-Aid. |
| 78 | Kelly | Canadian Institute of Health Research (160 Elgin Street, 9th Floor, Address Locator 4809A, Ottawa ON K1A 0 W9, Canada; Telephone: 613–941-2672) HIV Implementation Science – Component I funding opportunity [Funding Reference Number (FRN): 145372] |
| 38 | Kherghehpoush | Not disclosed AND conflict of interests not declared |
| 46 | Kirby | Funding Not disclosed |
| 45 | Kitutu | WHO Alliance for Health Policy and Systems Research, Einhorn Family Foundation, Pehr Lagermans Family, Sweden, Swedish Science Council and Uppsala University |
| 36 | Klepser | MEK, DGK, AMD-A, and SAK are codevelopers of the Community PharmacyeBased Point-of-Care Testing Certificate Program and receive paid honoraria or royalties for presentations and consulting. AMD-A is an employee of Walgreens and has received research funding from National Association of Chain Drug Stores (NACDS) Foundation, Quidel, Ferris State University College of Pharmacy, and Nebraska Department of Health. DGK has received research funding from the NACDS Foundation, State of Nebraska, State of Maryland, and Ferris State University and is a consultant for Arkray. MEK has received research funding from NACDS Foundation and is a member of the Cubist speakers bureau. SAK has received research funding from NACDS Foundation. Funding:  Funded by a grant from the National Association of Chain Drug Stores Foundation |
| 23 | Klepser | DG Klepser, ME Klepser, and AM Dering-Anderson are original developers of the point-of-care testing certificate program and receive honoraria and royalties for presentations and consulting. DG Klepser receives research funding from the National Association of Chain Drug Stores (NACDS), NACDS Foundation, Roche Diagnostics, and the State of Nebraska. ME Klepser receives research funding from the NACDS Foundation, Arkray, Roche Diagnostics, and SpartanBio. AM Dering-Anderson receives research funding from NACDS Foundation, Quidel, and the Nebraska Department of Health.  This research was supported by a generous grant from the NACDS Foundation, a 501(c) (3) not-for-profit charitable organization. NACDS Foundation funds were used to conduct research consistent with, and to advance, its charitable purpose to improve patient health through partnership in research, education and medication management |
| 52 | Klepser | Disclosure: Donald G. Klepser, Michael E. Klepser, and Keith M. Olsen are codevelopers of the Community PharmacyeBased Point-of-Care Testing Certificate Program and receive paid honoraria and royalties for presentations and consulting. Donald G. Klepser and Michael E. Klepser have received money for consulting services from Roche Diagnostics. The other authors declare no relevant conflicts of interest or financial relationships. Funding: This research was funded in part by a grant from Roche Diagnostics. |
| 39 | Kwarteng | Financial support: The study was funded by the Global Fund through the Ghana National Malaria Control Program of the Ghana Health Service. |
| 24 | Maloney | UK Department for International Development. |
| 40 | Mboyne | ACT Consortium, funded through a grant from the Bill and Melinda Gates Foundation to the London School of Hygiene and Tropical Medicine (grant number 39640). SC is supported by the Welcome Trust through a Research Career Development Fellowship (084933). |
| 41 | O' Meara | award number R01AI110478 from the National Institute of Allergy and Infectious Diseases of the National Institutes of Health (USA) |
| 26 | O'Connor | funding from the Idaho Board of Pharmacy, State of Idaho, and the CARES Act. |
| 50 | Papastergiou | No external funding |
| 51 | Poyer | Unitaid (data collection in Kwale County) and the UK Department for International Development (DFID) (data collection in Mombasa and Kilif counties) |
| 35 | Sim | J M O’Hara Research Fund managed by the Pharmaceutical Society of Western Australia. |
| 48 | Simmalavong | Global Fund to Fight AIDS, Tuberculosis, and Malaria, and technical assistance provided by WHO. |
| 42 | Sudhinaraset | Funding not disclosed but no competing interests declared |
| 43 | Thet | Alliance for Health Policy and Systems Research |
| 34 | Thornley | Boots UK but did not receive any payment or stock options, National Institute for Health Research University College London Hospitals Biomedical Research Centre. |
| 33 | Onwunduba | Royal Society of Tropical Medicine and Hygiene, UK, in partnership with the National Institute for Health and Care Research, UK |
| 47 | Soniran | WHO Special Programme for Research and Training in Tropical Diseases (TDR) Postdoctoral Fellowship programme in Implementation Research |
| 44 | Shelus | US Department of State, Bureau of Educational and Cultural Affairs [Fulbright-Fogarty Award in Public Health E0636820 to VS], the Doris Duke Charitable Foundation [Caregivers at Carolina Award 2015213 to RB], and the National Institutes of Health |
